# Polymorphisms in mTOR and Calcineurin Signaling Pathways Are Associated With Long-Term Clinical Outcomes in Kidney Transplant Recipients

**DOI:** 10.3389/fphar.2018.01296

**Published:** 2018-11-14

**Authors:** Antony Brayan Campos-Salazar, Fabiana Dalla Vecchia Genvigir, Claudia Rosso Felipe, Helio Tedesco-Silva, José Medina-Pestana, Gabriela Vieira Monteiro, Rodrigo de Gouveia Basso, Alvaro Cerda, Mario Hiroyuki Hirata, Rosario Dominguez Crespo Hirata

**Affiliations:** ^1^School of Pharmaceutical Sciences, University of São Paulo, São Paulo, Brazil; ^2^Bioinformatics and Pharmacogenetics Laboratory, METOSMOD Research Group, School of Pharmacy and Biochemistry, Universidad Nacional Mayor de San Marcos, Lima, Peru; ^3^Nephrology Division, Hospital do Rim, Federal University of São Paulo, São Paulo, Brazil; ^4^Department of Basic Sciences, Center of Excellence in Translational Medicine, BIOREN, Universidad de La Frontera, Temuco, Chile

**Keywords:** kidney transplant, immunosuppressive drugs, mTOR, calcineurin, FOXP3, pharmacogenetics

## Abstract

Monitoring of immunosuppressive drugs, such as calcineurin and mTOR inhibitors, is essential to avoid undesirable kidney transplant outcomes. Polymorphisms in pharmacokinetics-related genes have been associated with variability in blood levels of immunosuppressive drugs and adverse effects, but influence of pharmacodynamics-related genes remains to be elucidated. The influence of polymorphisms in genes of the mTOR and calcineurin signaling pathways on long-term clinical outcomes was investigated in Brazilian kidney transplant recipients within the 1-year post-transplant. Two-hundred and sixty-nine kidney transplant recipients were enrolled at a kidney transplant center in São Paulo city, Brazil, and treated with tacrolimus plus everolimus or mycophenolate sodium (clinical trial NCT01354301). Clinical and laboratory data, including renal function parameters and drug blood levels were recorded. Genomic DNA was extracted from blood samples. Polymorphisms in *MTOR* rs1057079 (c.4731G>A), rs1135172 (c.1437T>C), and rs1064261 (c.2997C>T); *PPP3CA* rs3730251 (c.249G>A); *FKBP1A* rs6033557 (n.259+24936T>C); *FKBP2* rs2159370 (c.-2110G>T); and *FOXP3* rs3761548 (c.-23+2882A>C) and rs2232365 (c.-22-902A>G) were analyzed by real-time PCR. Frequencies of gene polymorphisms did not differ among the treatment groups. Analysis of primary outcomes showed that patients carrying *MTOR* c.1437CC and *FOXP3* c.-23+2882CC genotypes had higher serum creatinine than non-carriers (*p* < 0.05) at 1-year post-transplant. *MTOR* c.4731G allele (AG+GG genotype) was associated with increased risk for acute rejection (OR = 3.53, 95% CI = 1.09–11.48, *p* = 0.037). Moreover, 1-year cumulative incidence of rejection was higher in *MTOR* c.4731G allele carriers compared to AA genotype carriers (*p* = 0.027). Individually, analysis of secondary outcomes revealed that *FKBP2* c.-2110GG genotype carriers had higher risk of leukopenia, *FKBP1A* n.259+24936C allele carriers had increased risk of constipation, and *FOXP3* c.-22-902A or c.-23+2882A allele had higher risk of gastrointestinal disorders (*p* < 0.05). However, these results were not maintained in the multivariable analysis after *p*-value adjustment. In conclusion, variants in genes of mTOR and calcineurin pathways are associated with long-term impaired renal function, increased risk of acute rejection, and, individually, with adverse events in Brazilian kidney transplant recipients.

## Introduction

The calcineurin inhibitor (CNI), such as tacrolimus (TAC) and mTOR inhibitor (mTORi), such as everolimus (EVR) are immunosuppressive drugs helpful to prevent allograft rejection and simultaneously improve graft and patient survival in kidney transplantation (Lim et al., 2017).

TAC and EVR binding to their cytoplasmic protein receptor, the FK506-binding protein 12 (FKBP12). The TAC-FKBP12 complex interacts with calcineurin while the EVR-FKBP12 targets mTOR. These complexes lead to inhibition of T cell activation and proliferation, besides other important implications in alloimmune responses (Tong and Jiang, 2015; Bergan et al., 2016).

The therapeutic monitoring of these immunosuppressants is widely accepted because there is high between-patient variability in their pharmacokinetics and concentration–effect relationship (van Gelder, 2014). In this way, in addition to biodemographic and clinical factors, genetics play an important role. The key genetic variants involved in the metabolism and distribution of the immunosuppressive drugs have been largely investigated (Picard et al., 2016; Zhang et al., 2018). On the contrary, those involved with drug targets and molecular signaling pathway remain poorly known in solid organ transplantation.

In kidney transplant recipients on CNI-based immunosuppression, some polymorphisms in three calcineurin subunit genes (*PPP3CA*, *PPP3CB*, and *PPP3R1*) were not associated with renal function and incidence of rejection or adverse events (Moes et al., 2016; Pouché et al., 2016a; Salgado et al., 2017).

Five variants in *MTOR* (rs1770345, rs2300095, rs2076655, rs1883965, and rs12732063) were investigated in kidney transplant recipients switched from CNI to mTORi. The AGAAA haplotype was associated with a slightly decreasing in hemoglobin levels (Woillard et al., 2012), but it seems that it was due to the physiological mechanism rather than pharmacogenetic (Pouché et al., 2016b).

This study investigated the influence of polymorphisms in *MTOR*, *PPP3CA*, *FKBP1A* (FKBP12), *FKBP2*, and *FOXP3* on long-term clinical outcomes (renal function, acute rejection and adverse events) of kidney recipients treated with TAC and EVR-based immunosuppressive therapy. *FKBP2* encodes the FKBP13 that is closely related to FKBP12 (Tong and Jiang, 2015). FOXP3 is a key transcriptional factor of the regulatory T cells (Tregs), which are important in suppressing alloimmune responses and maintenance of transplantation tolerance (Dummer et al., 2012).

## Materials and Methods

### Study Population

This pharmacogenetic study was carried out in a sample of kidney transplant recipients previously enrolled in the clinical trial registered as NCT01354301 at the US Clinical Trials database (Tedesco-Silva et al., 2015). The study was approved by the Ethics Committee of the UNIFESP (Protocol # 0339/11) and performed according to the international regulations in Good Clinical Practices and to the ethical principles of the Declaration of Helsinki. All patients provided written informed consent prior to enrollment.

The aforementioned trial included low/moderate-immunological risk adult recipients indicated for first ABO-compatible kidney transplantation, from either alive or deceased donor, of which, the 269 patients who completed the study were selected. Exclusion criteria considered kidneys from HLA identical or expanded criteria deceased donors, positive cytotoxic cross match or panel reactive antibody equal to or above 50%, either class I or class II. Use of contraceptives methods during the trial were requested for women of childbearing potential (Tedesco-Silva et al., 2015).

### Immunosuppressive Regimens

The patients were randomized in three study groups of immunossupressive treatment, as follows: (i) Group TAC5/EVR: single dose of anti-thymocyte globulin at first day post-transplant, TAC 0.05 mg/kg b.i.d. and EVR 1.5 mg daily b.i.d.; (ii) TAC10/EVR: basiliximab induction on days 0 and 4. On day 1, TAC 0.1 mg/kg b.i.d. and EVR 1.5 mg b.i.d.; (iii) TAC10/MPS: basiliximab induction on days 0 and 4. On day 1, TAC 0.1 mg/kg b.i.d. and MPS 720-mg b.i.d. Before transplantation, all patients were treated with 1 g of methylprednisolone, and began receiving on day 1 post-transplant 0.5 mg/kg/day oral prednisone (not exceeding a 30 mg dose) tapered to 5 mg/day dose by day 45.

### Clinical Characteristics and Laboratory Data

Clinical characteristics and laboratory data of the kidney recipients were recorded pre- and post-transplant). Clinical characteristics included cause of CKD, dialysis procedure, maintenance hemodialysis, cold ischemia time, graft loss and DGF. Laboratory data consisted of serum creatinine, eGFR, glucose, insulin, total cholesterol, HDL and LDL cholesterol, triglycerides, hemoglobin, erythrocytes and leukocyte counts, urinary proteins, immunodiagnostic test for CMV, kidney graft biopsies. eGFR was estimated by MDRD formula (Levey et al., 2009).

### Immunosuppressive Drug Monitoring

TAC and EVR blood concentration, the prescribed doses and adjusted concentration for dose administered (Co/D) were recorded. Blood concentrations were analyzed by chemoluminiscent microparticle immunoassay (Abbott Diagnostics, Lake Forest, IL, United States) and liquid chromatography-tandem mass spectrometry, respectively.

### Clinical Outcomes

Primary clinical outcomes were considered serum creatinine, eGFR, cellular and humoral acute kidney rejection, and CMV events, including asymptomatic (infection) or symptomatic (disease) viremia (Kotton et al., 2013).

Secondary clinical outcomes were considered the adverse events that were classified according to the Medical Dictionary for Regulatory activities (Medical Dictionary for Regulatory Activities [MedDRA], 2016), as: blood and lymphatic system disorders, gastrointestinal disorders, general disorders and administration site conditions, metabolism and nutrition disorders, and renal and urinary disorders. Recurrence of adverse event was not considered in the analysis of the secondary outcomes.

### Genomic DNA Extraction

Genomic DNA was isolated from peripheral blood sample using the QIAamp^®^ DNA Blood Mini Kit (Qiagen Inc., Valencia, CA, United States) on the QIAcube system (Qiagen Sciences Inc., Germantown, MD, United States). DNA quantification and purity were evaluated by 260/280 nm spectrophotometry using NanoDrop ND-1000 (NanoDrop Technologies Inc., Wilmington, NC, United States) and DNA integrity was evaluated by 1.0% agarose gel electrophoresis. Samples were stored at −20°C.

### Gene Polymorphisms Selection and Analysis

Gene polymorphisms [*MTOR* rs1057079 (c.4731G>A, p.Ala1577=), rs1135172 (c.1437T>C, p.Asp479=) and rs1064261 (c.2997C>T, p.Asn999=); *PPP3CA* rs3730251 (c.249G>A, p.Ala83=); *FKBP1A* rs6033557 (n. 259+24936T>C); *FKBP2* rs2159370 (c.-2110G>T); and *FOXP3* rs2232365 (c.-22-902A>G), and rs3761548 (c.-23+2882A>C)] were selected using a two-step approach based on pathway-gene method, as described elsewhere (Kooloos et al., 2009).

Genotyping was performed using the following pre-designed TaqMan SNP genotyping assays (Thermo Fisher Scientific Inc., Foster City, CA, United States): C_8862305_1 (rs1057079), C_8862346_10 (rs1135172), C_28027381_10 (rs1064261), C_25618069_10 (rs3730251), C__30149598_20 (rs6033557), C___2981262_20 (rs2159370), C__15942641_10 (rs2232365), C__27476877_10 (rs3761548). The PCR mixture contained 1X TaqMan genotyping master mix, 1X TaqMan SNP genotyping assay and 40 ng of template DNA. The real-time PCR assays were carried out according to the manufacturer’s instructions using an ABI 7500 FAST system (Applied Biosystems, Foster City, CA, United States). Genotype calling was performed using 7500 SDS software (Thermo Fisher Scientific Inc., Foster City, CA, United States). To assess the accuracy of the genotype calling, 20% of the DNA samples were randomly genotyped twice.

### Statistical Analysis

Continuous variables are expressed as median and interquartile range and compared by Mann–Whitney or Kruskal–Wallis test and Bonferroni’s correction test for multiple comparisons. Categorical variables are expressed as percentage and number of individuals in parenthesis, and compared by chi-square test or likelihood ratio test (for number of individuals less than 5).

Allele and genotype frequencies were estimated by counting. Hardy–Weinberg equilibrium (HWE) was estimated using Haploview program (Barrett et al., 2005). For polymorphisms located at X chromosome HWE was estimated considering only female individuals in order to avoid hemizygous male genotypes.

The influence of gene polymorphisms on clinical outcomes was studied using dominant, co-dominant and recessive inheritance models. The dominant model, in which the presence of at least one minor allele has an effect on the phenotype or clinical outcome, provides the highest power values of the statistical tests (Hong and Park, 2012). Therefore, homozygous and heterozygous patients carrying the minor allele were analyzed together.

Cumulative incidence of acute rejection episodes (time-dependent variable) during the follow-up was analyzed by Kaplan–Meier survival analysis, and comparisons between major and minor allele carriers were performed using the log-rank test.

Univariable logistic regression analysis was used to assess the relationship between gene variants and primary and secondary clinical outcomes. Continuous variables such as eGFR and serum creatinine were divided by tertiles to be used as categorical variables in the logistic regression analysis. The combined effect of covariates and SNP was investigated using multivariable logistic regression analysis. The covariates considered in the models were age, weight, gender, donor type, time on hemodialysis, DGF time, cold ischemia time, acute rejection, proteinuria, immunosuppressive therapy group and CMV. Only covariates with *p*-value lower than 0.15 in univariable logistic regression analysis were included in the multivariable logistic regression (Heinze and Dunkler, 2017). To account for multiple hypothesis testing in model with more than two covariates, adjustment of *p*-values was performed using Bonferroni–Holm sequential correction method.

Association analysis of *MTOR* polymorphisms with primary and secondary outcomes was carried out with data from groups treated with EVR (TAC5/EVR and TAC10/EVR), which is the mTORi in the immunosuppressive therapy.

The statistical analyses were performed using the SPSS for windows (SPSS Inc., Chicago, IL, United States), GraphPad Prism (GraphPad Software Inc., La Jolla, CA, United States) and SigmaStat (Systat Software Inc., San Jose, CA, United States). The *p*-value threshold for significance was 0.05.

## Results

### Characteristics of the Study Population

Biodemographic and clinical characteristics of the kidney transplant recipients are shown in Table 1. Median age and weight of the kidney recipients were 44.0 [34.0–56.0] years and 68.3 [59.0–77.9] kg, respectively. The studied group had mainly men (66.2%) and Caucasians (51.7%). Other self-reported ethnicities were African (14.9%) and intermediate/mixed (33.4%). Causes of CKD were glomerulonephritis (12.3%), hypertension (10.0%) and diabetes (10.0%). Hemodialysis was the most frequent dialysis procedure (93.3%) lasting a median time of 33.0 [16.0–54.0] months. Grafts were exposed to cold ischemia for 20.3 [17.6–23.0] h. DGF was observed in 33.8% of the kidney recipients, and it lasted 10.0 [6.0–15.0] days. Kidney grafts were mainly obtained from deceased donors (69.1%). The proportion of these variables did not differ among the therapy groups, except for graft loss which was higher in the TAC10/MPS group (7.5%) compared to TAC5/EVR (1.3%) and TAC10/EVR (1.0%) (*p* = 0.026).

**Table 1:** Biodemographic and pre-transplant clinical data of kidney recipients.

Variables	Total (269)	TAC5/EVR (80)	TAC10/EVR (96)	TAC10/MPS (93)	*p*-Value
Recipient age, years	44.0 [34.0–56.0]	43.5 [32.3–55.8]	46.0 [33.5–57.0]	43.0 [35.0–55.5]	0.667
Recipient weight, kg	68.3 [59.0–77.9]	68.8 [62.4–77.5]	67.5 [58.1–76.9]	68.5 [57.3–79.8]	0.652
Recipient gender, male	66.2% (178)	63.8% (51)	65.6% (63)	68.8% (64)	
Recipient ethnics, Caucasian	51.7% (139)	47.5% (38)	51.0% (49)	55.9% (52)	0.386
Cause of CKD					0.260
Glomerulonephritis	12.3% (33)	13.8% (11)	14.6% (14)	8.6% (8)	
Hypertension	10.0% (27)	10.0% (8)	7.3% (7)	12.9% (12)	
Diabetes	10.0% (27)	8.8% (7)	5.2% (5)	16.1% (15)	
Undetermined	41.6% (112)	41.3% (33)	46.9% (45)	36.6% (34)	
Other	26.0% (70)	26.3% (21)	26.0% (25)	25.8% (24)	
Hemodialysis	93.3% (251)	91.3% (73)	95.8% (92)	92.5% (86)	0.586^∗^
Time on hemodialysis, months	33.0 [16.0–54.0]	36.0 [16.0–48.0]	27.5 [16.3–49.8]	29.5 [16.0–61.5]	0.894
Cold ischemia time^a^, h	20.3 [17.6–23.0]	20.3 [17.6–23.7]	20.5 [17.8–23.0]	20.1 [17.2–23.0]	0.839
Delayed graft function	33.8% (91)	37.5% (30)	34.4% (33)	30.1% (28)	0.586
DGF time, days	10.0 [6.0–15.0]	12.0 [6.8–16.0]	11.0 [6.0–14.0]	9.0 [5.3–13.5]	0.396
Graft loss	3.3% (9)	1.3% (1)	1.0% (1)	7.5% (7)	0.026^∗^
Donor type, deceased	69.1% (186)	77.5% (62)	64.6% (62)	66.7% (62)	0.148

*Categorical variables are shown as percentage and number of individuals in parenthesis and compared by chi-square or likelihood ratio (^∗^) tests. Continuous variables are shown as median [interquartile range] and compared by Kruskal–Wallis test followed by Bonferroni test for multiple comparisons. ^a^Cold ischemia time was estimated for grafts received from deceased donors. CKD, chronic kidney disease; DGF, delayed graft function.*

Drug monitoring data of the kidney recipients at 1-year post-transplant is shown in Supplementary Table 1. TAC concentration was higher in TAC10/MPS group compared to TAC5/EVR and TAC10/EVR groups (<0.05), while TAC Co/Do were similar among the therapy groups. EVR monitoring data did not differ between the TAC5/EVR and TAC10/EVR groups (*p* > 0.05).

### Clinical Outcomes

Primary and secondary kidney outcomes at 1-year post-transplant are shown in Table 2. In the total group, serum creatinine and eGFR were 1.3 [1.1–1.6] mg/dL and 63.5 [49.7–78.2] ml/min/1.73 m^2^, respectively. TAC10/EVR group had higher serum creatinine and lower eGFR compared to TAC10/MPS group (*p* < 0.05). Acute rejection occurred in 14.9% of the patients, mainly T cell-mediated episodes (13.4%), but it did not differ among the treatment groups (*p* = 0.198).

**Table 2:** Primary and secondary clinical outcomes of kidney recipients at 1-year post-transplant.

Variables	Total (269)	TAC5/EVR (80)	TAC10/EVR (96)	TAC10/MPS (93)	*p*-Value
**Primary outcomes**					
Serum creatinine, mg/dL	1.3 [1.1–1.6]	1.3 [1.1–1.7]^a,b^	1.4 [1.1–1.8]^a^	1.2 [1.0–1.5]^b^	0.034
eGFR, ml/min/1.73 m^2^	63.5 [49.7–78.2]	64.6 [48.9–79.1]^a,b^	57.8 [44.6–70.7]^a^	67.3 [55.7–81.5]^b^	0.014
Acute rejection,	14.9% (40)	10.0% (8)	17.7% (17)	16.2% (15)	0.198
Cellular	13.4% (36)	7.5% (6)	17.7% (17)	14.0% (13)	
Humoral	1.5% (4)	2.5% (2)	0.0% (0)	2.2% (2)	
**Secondary outcomes**					
*Blood and lymphatic system disorders*					
Anemia	8.2% (22)	10.0% (8)	9.4% (9)	5.4% (5)	0.470
Leukopenia	5.2% (14)	5.0% (4)	1.0% (1)	9.7% (9)	0.018^∗^
**Gastrointestinal disorders**					
Constipation	12.6% (34)	11.3% (9)	10.4% (10)	16.1% (15)	0.450
Diarrhea	25.7% (69)	25.0% (20)	22.9% (22)	29.0% (27)	0.621
Dyspeptic disorder	8.6% (23)	3.8% (3)	9.4% (9)	11.8% (11)	0.156
Epigastric pain	12.3% (33)	11.3% (9)	9.4% (9)	16.1% (15)	0.348
Nausea and/or vomiting	9.7% (26)	8.8% (7)	10.4% (10)	9.7% (9)	0.933
**General disorders and administration site conditions**					
Edema	41.3% (111)	45.0% (36)	47.9% (46)	31.2% (29)	0.047
**Infections and infestations**					
Cytomegalovirus	18.2% (49)	5.0% (4)	10.4% (10)	37.6% (35)	<0.001^∗^
**Metabolism and nutritional disorders**					
Hyperglycemia	17.8% (48)	25.0% (20)	15.6% (15)	14.0% (13)	0.131
Post-transplant diabetes	10.4% (28)	13.8% (11)	11.5% (11)	6.5% (6)	0.268
**Renal and urinary disorders**					
Proteinuria	8.9% (24)	10.0% (8)	9.4% (9)	7.5% (7)	0.835

*Categorical variables are shown as percentage and number of individuals in parenthesis and compared by chi-square or likelihood ratio (^∗^) tests. Continuous variables are shown as median [interquartile range] and compared by Kruskal–Wallis test and Bonferroni test for multiple comparisons. Different superscript letters indicate significant differences. eGFR, estimated glomerular filtration rate.*

Analysis of the secondary outcomes showed that the most common blood and lymphatic system disorders were anemia (8.2%) and leucopenia (5.2%). Leukopenia was more frequent in the TAC10/MPS group (9.7%) than the TAC5/EVR (5.0%) and TAC10/EVR (1.0%) groups (*p* = 0.018) (Table 2). Gastrointestinal disorders were mainly constipation (12.6%), diarrhea (25.7%), dyspeptic disorder (8.6%), epigastric pain (12.3%) and nausea and/or vomiting (9.7%), but frequencies of these events were similar among the therapy groups (*p*. > 0.05). Edema was the most common general disorder (41.3%) with the higher proportion in TAC5/EVR (45.0%) and TAC10/EVR (47.9%) groups than in the TAC10/MPS (31.2%) (*p* = 0.047). CMV infection and/or disease was detected in 18.2% of the total group, but it was more frequent in TAC10/MPS group (37.6%) compared with the other groups (*p* < 0.001). Hyperglycemia (17.8%) and post-transplant diabetes (10.4%) were the common metabolism and nutritional disorders. Proteinuria, a renal and urinary disorder, was detected in 8.9% of the total kidney recipients.

### Gene Polymorphisms and Primary Clinical Outcomes

Frequencies of gene polymorphisms in the sample population are summarized in Table 3. Genotypes and minor allele frequencies of the SNP were similar among the study groups (*p* > 0.05). Further, no deviation from HWE was observed (data not shown). These results are suggestive that patients were randomly distributed in the therapy groups.

**Table 3:** Frequencies of gene polymorphisms in kidney transplant recipients.

Polymorphism	Genotypes	Total (269)	TAC5/EVR (80)	TAC10/EVR (96)	TAC10/MPS (93)	*p*-Value
*MTOR* rs1135172	CC	33.5% (90)	32.5% (26)	39.6% (38)	28.0% (26)	0.534
c.1437T>C	CT	47.2% (127)	46.3% (37)	43.8% (42)	51.6% (48)	
	TT	19.3% (52)	21.3% (17)	16.7% (16)	20.4% (19)	
	T allele	42.9%	44.4%	38.5%	46.2%	0.290
*MTOR* rs1064261	TT	42.4% (114)	35.0% (28)	46.9% (45)	44.1% (41)	0.530
*c.*2997C>T	TC	45.7% (123)	50.0% (40)	43.8% (42)	44.1% (41)	
	CC	11.9% (32)	15.0% (12)	9.4% (9)	11.8% (11)	
	C allele	34.8%	40.0%	31.3%	33.9%	0.218
*MTOR* rs1057079	AA	33.1% (89)	30.0% (24)	40.6% (39)	28.0% (26)	0.138
c.4731G>A	AG	43.9% (118)	40.0% (32)	39.6% (38)	51.6% (48)	
	GG	23.0% (62)	30.0% (24)	19.8% (19)	20.4% (19)	
	G allele	45.0%	50.0%	39.6%	46.2%	0.135
*PPP3CA* rs3730251	GG	85.5% (230)	86.3% (69)	87.5% (84)	82.8% (77)	0.481^∗^
c.249G>A	GA	14.1% (38)	12.5% (10)	12.5% (12)	17.2% (16)	
	AA	0.4% (1)	1.3% (1)	0.0% (0)	0.0% (0)	
	A allele	7.4%	7.5%	6.3%	8.6%	0.684^∗^
*FKBP1A* rs6033557	TT	43.9% (118)	37.5% (30)	46.9% (45)	46.2% (43)	0.209
n.259+24936T>C	TC	44.6% (120)	48.8% (39)	46.9% (45)	38.7% (36)	
	CC	11.5% (31)	13.8% (11)	6.3% (6)	15.1% (14)	
	C allele	33.8%	38.1%	29.7%	34.4%	0.244
*FKBP2* rs2159370	GG	33.5% (90)	32.5% (26)	34.4% (33)	33.3% (31)	0.997
c.-2110G>T	GT	52.0% (140)	53.8% (43)	51.0% (49)	51.6% (48)	
	TT	14.5% (39)	13.8% (11)	14.6% (14)	15.1% (14)	
	T allele	40.5%	40.6%	40.1%	40.9%	0.988
*FOXP3* rs2232365	GG	52.8% (142)	51.3% (41)	55.2% (53)	51.6% (48)	0.721
c.-22-902A>G	GA	18.2% (49)	16.3% (13)	20.8% (20)	17.2% (16)	
	AA	29.0% (78)	32.5% (26)	24.0% (23)	31.2% (29)	
	A allele	38.1%	40.6%	34.4%	39.8%	0.426
*FOXP3* rs3761548	CC	65.1% (175)	67.5% (54)	63.5% (61)	64.5% (60)	0.666
c.-23+2882A>C	CA	13.0% (35)	15.0% (12)	10.4% (10)	14.0% (13)	
	AA	21.9% (59)	17.5% (14)	26.0% (25)	21.5% (20)	
	A allele	28.4%	25.0%	26.0%	28.5%	0.433

*Genotype and minor allele frequencies are shown as percentage and number of individuals in parenthesis, and compared by chi-square or likelihood ratio (^∗^) tests.*

**FIGURE 1:**
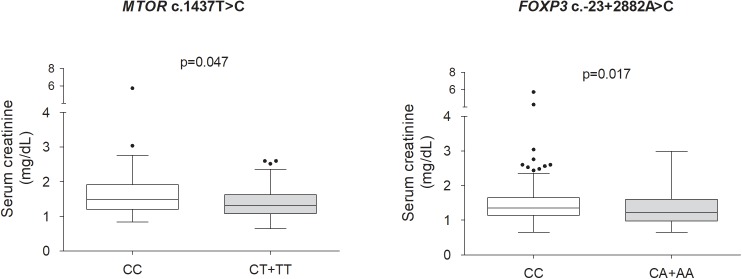
Influence of *MTOR* and *FOXP3* variants on serum creatinine in kidney recipients at 1-year post-transplant. Data are shown as median and interquartile range, and were compared by Mann–Whitney test. Comparisons between *MTOR* c.1437T>C genotypes were carried with data from TAC5/EVR and TAC10/EVR groups.

Median values of serum creatinine at 1-year post-transplant were higher in subjects carrying *MTOR* c.1437T>C CC genotype and *FOXP3* c.-23+2882A>C CC genotype (Figure 1). The association of gene polymorphisms with the pre-transplant levels of serum creatinine was further investigated, and no differences were found between carriers of the different genotypes of *MTOR* (Supplementary Figure 1). However, individuals carrying the *FOXP3* c.-23+2882CC genotype had higher values of pre-transplant serum creatinine than A allele (genotype CA+AA) carriers (Supplementary Figure 2). The studied polymorphisms did not influence eGFR values (data not shown). We also investigated the influence of *MTOR* c.1437T>C and *FOXP3* c.-23+2882A>C variants on TAC blood concentrations at month 12 but no significant differences were found between genotype carriers (*p* > 0.05) (data not shown). This result is suggestive that immunosuppressant concentration did not influence in the association between genetic variants and serum creatinine.

Univariable logistic regression showed that *FOXP3* c.-23+2882CC genotype was associated with increased serum creatinine at 1-year post-transplant (OR = 1.75, 95% CI = 1.07–2.86, *p* = 0.025) (Supplementary Table 2). Patient weight and gender and acute rejection were also variables associated with increased risk for high serum creatinine (*p* < 0.05), independently of the groups therapy tested (models 1 and 2) (Supplementary Table 3).

Multivariable regression analysis revealed that male gender remained a risk factor for high serum creatinine at 1-year post-transplant using both model 1 (TAC5/EVR and TAC10/EVR) and model 2 (all therapy groups) (adjusted *p* < 0.05), while acute rejection was confirmed only using the model 2 (adjusted *p* = 0.024) (Table 4).

**Table 4:** Variables associated with primary outcomes in kidney recipients at 1-year post-transplant: Multivariable logistic regression analysis.

Dependent variables	Independent variables	Risk factor	OR (95% CI)	*p*-Value	Adjusted *p*-value
**High serum creatinine**					
Model 1	*MTOR* c.1437T>C	CC genotype	1.66 (0.86–3.32)	0.104	0.312
	Weight	Per 1 kg	1.01 (0.99–1.04)	0.325	0.442
	Gender	Male	2.97 (1.46–7.49)	0.001	0.005
	Acute rejection	Presence	3.51 (1.35–13.04)	0.015	0.060
	Proteinuria	Presence	1.72 (0.69–4.85)	0.221	0.442
Model 2	*FOXP3* c.-23+2882A>C	CC genotype	1.22 (0.69–2.22)	0.465	0.516
	Age	Per 1 year	0.99 (0.97–1.00)	0.112	0.448
	Weight	Per 1 kg	1.02 (0.99–1.04)	0.172	0.516
	Gender	Male	3.67 (1.96–7.80)	0.0001	0.001
	Acute rejection	Presence	3.74 (1.71–10.40)	0.004	0.024
	Proteinuria	Presence	1.71 (0.73–4.55)	0.226	0.516
	Therapy group	TAC+EVR	1.82 (1.05–3.31)	0.019	0.095
**Acute rejection episodes**					
Model 1	*MTOR* c.4731G>A	AG+GG genotype	3.53 (1.09–11.48)	0.037	0.111
	Age	Per 1 year	0.97 (0.94–1.01)	0.130	0.260
	Weight	Per 1 kg	1.03 (0.99–1.06)	0.144	0.260
	Delayed graft function time	Per 1 day	1.09 (1.03–1.15)	0.003	0.012
Model 2	Age	Per 1 year	0.98 (0.95–1.00)	0.072	0.072
	Delayed graft function time	Per 1 day	1.11 (1.06–1.16)	0.0001	0.0001

*Model 1: Analysis for MTOR polymorphisms was carried out with data from TAC5/EVR and TAC10/EVR groups. Model 2: Analysis for all polymorphisms except MTOR was performed with data from the TAC5/EVR, TAC10/EVR and TAC10/MPS groups. Adjusted p-value was estimated using the Bonferroni–Holm sequential correction method. OR, odds ratio; CI, confidence interval.*

Association of genetic and non-genetic variables associated with acute rejection within 1-year post-transplant was also tested by univariate regression analysis (Supplementary Table 4). *MTOR* c.4731G allele (AG+GG genotype) carriers had increased risk for acute rejection (OR = 3.37, 95% CI = 1.10–10.30, *p* = 0.037). Moreover, patients with increased DGF time have higher risk of acute rejection using both model 1 (*p* = 0.001) and model 2 (*p* = 0.0001).

Multivariable regression analysis showed that *MTOR* c.4731G allele remained associated with acute rejection (*p* = 0.037), but not after Bonferroni–Holm correction (adjusted *p* = 0.111), whereas increased DGF time was confirmed as a risk factor for rejection (adjusted *p* < 0.05) (Table 4).

**FIGURE 2:**
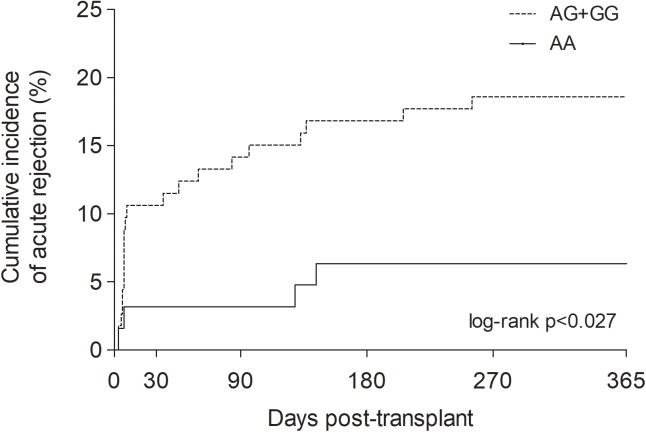
One-year cumulative incidence of acute rejection in kidney recipients stratified by *MTOR* c.4731G>A genotypes. Log-rank *p*-value was estimated by Kaplan–Meier survival analysis.

The influence of *MTOR* c.4731G>A on 1-year cumulative incidence of acute rejection was further investigated using the Kaplan–Meier survival analysis. Patients carrying the G allele (AG+GG genotype) had higher cumulative incidence of acute rejection (18.6%) compared to AA genotype carriers (6.3%; log-rank *p* < 0.027) (Figure 2).

### Gene Polymorphisms and Secondary Clinical Outcomes

The results of the univariable regression analysis for gene polymorphism and other variables associated with secondary clinical outcomes within 1-year post-transplant of kidney recipients are shown in Supplementary Table 5. *FKBP2* c.-2110GG genotype (OR = 3.87, 95% CI = 1.26–11.91, *p* = 0.018), therapy group (TAC+MPS; OR = 3.66, 95% CI = 1.19–11.28, *p* = 0.024) and CMV (OR = 9.68, 95% CI = 3.08–30.38, *p* = 0.0001) were associated with leukopenia (*p* < 0.05). *FKBP1A* n.259+24936C allele (TC+CC genotype) was associated with constipation (OR = 2.40, 95% CI = 1.08–5.37, *p* = 0.033). *FOXP3* c.-22-902A allele (GA+AA genotype) was associated with epigastric pain (OR = 2.15, 95% CI = 1.01–4.56, *p* = 0.047) and *FOXP3* c.-23+2882A allele (CA+AA genotype) was associated with nausea and/or vomiting episodes (OR = 2.38, 95% CI = 1.05–5.38, *p* = 0.038).

Multivariable regression analysis showed that *FKBP2* c.-2110GG genotype did not remained associated with leukopenia using the model 1 (adjusted *p* = 0.085) (Table 5). The *FKBP1A* and *FOXP3* variants were associated, respectively, with constipation and epigastric pain using the model 2 (*p* < 0.05), but these results were not confirmed after Bonferroni–Holm correction (adjusted *p* > 0.05).

**Table 5:** Variables associated with secondary outcomes in kidney recipients at 1-year post-transplant: Multivariable logistic regression analysis.

Dependent variable	Independent variable	Risk factor	OR (95% CI)	*p*-Value	Adjusted *p*-value
**Model 1**					
Leukopenia	*FKBP2* c.-2110G>T	GG genotype	4.45 (1.81–20.11)	0.017	0.085
	CMV	Use of ganciclovir	6.85 (1.81–26.02)	0.005	0.030
**Model 2**					
Constipation	*FKBP1A* n.259+24936T>C	TC+CC genotype	2.41 (1.05–7.92)	0.030	0.150
Epigastric pain	*FOXP3* c.-22-902A>G	GA+AA genotype	2.41 (1.05–6.33)	0.022	0.115
Nausea and/or vomiting	*FOXP3* c.-23+2882A>C	CA+AA genotype	2.24 (0.89–6.23)	0.063	0.189

*Model 1: Adjusted for recipient weight, age and gender, therapy group and treatment for cytomegalovirus. Model 2: Adjusted for recipient weight, age and gender and therapy group. Adjusted p-value was estimated using the Bonferroni–Holm sequential correction method. OR, odds ratio; CI, confidence interval.*

## Discussion

This study investigated the influence of polymorphisms in genes of the mTOR and calcineurin signaling pathway on long-term clinical outcomes in kidney recipients treated with TAC and EVR-based immunosuppressive therapy. Factors related to long-term acute rejection and adverse events were also investigated. Pre-transplant clinical and laboratory characteristics of the kidney recipients were similar among the immunosuppressive therapy groups, as it was previously reported (Tedesco-Silva et al., 2015). Interestingly the TAC10/EVR group had lower eGFR and higher serum creatinine than TAC10/MPS at 1-year post-transplant.

A meta-analysis examined long-term renal outcomes comparing different immunosuppressant regimens. The authors observed that cohorts using combination of non-reduced doses of CNI and mycophenolic acid, as compared with mTORi group, showed higher creatinine clearance and lower serum creatinine values (Xie et al., 2015). Therefore, in agreement to our results, the combination of traditional CNI regimens and MPS might lead to better renal function. In a large Brazilian single-center study, after 1 year, no differences in eGFR values were found between *de novo* use of mTORi or antimetabolites, such as mycophenolic acid, in combination with CNIs (de Paula et al., 2016), but a higher proportion of living donors could have influenced this result.

In this study, *MTOR* c.1437T>C (CC genotype) was associated with higher serum creatinine, but this variant did not influence eGFR at 1-year post-transplant. No previous study evaluated the relationship between *MTOR* variants and renal outcomes in kidney recipients. *MTOR* c.1437T>C (p.Asp479=) does not cause a functional disruption in the mTOR protein, which could be related to a worst renal outcome. It is conceivable that this variant is in linkage disequilibrium with a pathogenic variant within *MTOR*.

*MTOR* c.1437T>C was reported to be linked to the *MTOR* rs2295080 (c.-141C>A), a promoter variant, of which GT genotype was previously associated with less risk for prostate cancer (Liu et al., 2017). Further *in vitro* experiments with luciferase reporter in normal or cancer gastric cell lines (GES-1, BGC823, MGC803, and SGC-7901) revealed a higher transcription activity in the presence of the T allele of rs2295080 (Xu et al., 2013).

It is likely that the C allele of *MTOR* c.1437T>C is correlated with the T allele of *MTOR* rs2295080 (c.-141C>A), and the higher mTOR transcriptional levels might lead to a worst renal outcome due to insufficient immunosuppressive activity of EVR.

Carriers of the G allele of *MTOR* c.4731G>A (genotype AG+GG) were found to present higher incidence of acute rejection episodes during the first-year post-transplant. Regression analysis showed that G allele of the *MTOR* c.4731G>A polymorphism was associated with increased risk for long-term acute rejection in kidney transplants. This variant, which is in linkage disequilibrium with *MTOR* c.1787-116A>G, was previously reported to be associated with high risk for colon cancer in a sample from United States (Slattery et al., 2010). Also, the *MTOR* c.1787-116AA genotype was associated with reduced *MTOR* mRNA expression in healthy colon tissue (Slattery et al., 2014).

These evidences are suggestive that the subjects carrying the *MTOR* c.4731G allele may have lower *MTOR* transcript levels, which could be associated with increased incidence of acute rejection. Besides, the *MTOR* variants, mainly *MTOR* c.1787-116A>G (rs2024627), were recommended as genetic markers of pharmacogenetics of kidney transplant (Pouché et al., 2016b). Finally, patient variability within the efficacy kidney outcomes may rely on the *MTOR* variants yet to be studied.

Alloantigens are immunomodulatory molecules that stimulate T cell receptor (Ingulli, 2010); upon its activation, mTOR is activated, which in turn regulates T cell fate (Chi, 2012). Immunosuppressant agents targeting mTOR as EVR inhibit the transduction of interleukin-2 stimulus in T cells causing a cell-cycle arrest (Ma et al., 2018). On those alloactivated T cells, mTOR inhibitors might reduce the immune response toward the graft, avoiding acute rejection episodes through the reduction of mTOR levels.

mTOR signaling pathway also exerts a role regulating the differentiation of regulatory and effectors T cells (Zeng and Chi, 2017), and its activation led to the proliferation and suppressive activity of Tregs. In this way, inactivation of the mTOR pathway could also led to a lower activity of Tregs, and subsequent autoimmunity problems (Zeng et al., 2013). In agreement to our hypothesis, the lower expression of mTOR could turn the graft more vulnerable to immune responses, and subsequent acute rejection episodes. Therefore, in spite of mechanistic explanations for acute rejection and *MTOR* variants, the relevance of polymorphisms in the mTOR signaling pathway is pointed out by our study.

*MTOR* variants were not associated with long-term adverse events in this work, in spite of a previous study reported a relationship of other *MTOR* polymorphisms with low hemoglobin levels in kidney recipients treated with sirolimus-based immunosuppressive therapy (Woillard et al., 2012).

In this study, the CC genotype of the *FOXP3* c.-23+2882A>C was associated with higher serum creatinine pre- and post-transplant in kidney recipients. It is likely that *FOXP3* c.-23+2882CC genotype may account for a worse renal function. On the other hand, a previous study showed that individuals carrying the CC genotype had increased eGFR values and TAC blood levels in kidney recipients at 21 days post-transplant (Ge et al., 2016). These results are suggestive that the influence of this *FOXP3* variant on renal function may depend on the time of exposure to TAC.

Additional analysis used an additive model, which grouped male hemizygous genotypes with each one of the corresponding female homozygous genotypes. Our results indicated that females carrying the CA genotype, but not CC/C, of the *FOXP3* c.-23+2882A>C had a higher risk of serum creatinine compared to the individuals carrying the AA/A genotypes/alleles (data not shown). Subgroup analysis in small samples could lower statistical power. Besides, disparity in the frequency distribution of the female heterozygotes could also have influenced this result.

One study explored the impact of *FOXP3* polymorphisms on the long-term renal outcomes in patients receiving a cyclosporine-based treatment. *FOXP3* c.-22-2361C>T but not c.-23+2882A>C was associated with lower eGFR values. To our knowledge, this is the only study which explored *FOXP3* polymorphisms considering hemizygous males (Xu et al., 2018), and even though did not report any association for *FOXP3* c.-23+2882A>C polymorphism, it drew the attention to these group of genetic variants.

*FOXP3* c.-22-902A>G and c.-23+2882A>C polymorphisms were not related to long term acute rejection in kidney recipients, even when the analysis was carried out considering male as hemizygous and grouping with correspondent homozygous genotypes (data not shown). A previous study also did not find association of both *FOXP3* variant with acute or chronic rejection, even in a 10-years follow-up period (Park et al., 2017). On the other hand, other studies reported association of the *FOXP3* c.-23+2882A>C (A allele) polymorphism with long-term (2 and 5 years) acute rejection or nephrotoxicity in kidney recipients from different populations treated with cyclosporine- or TAC- based immunosuppressive therapy (Qiu et al., 2012; Misra et al., 2016; Wu et al., 2017). The c.-23+2882A allele was also associated with worst graft survival and higher rate of recurrence of the original glomerular disease in kidney recipients at 3-year post-transplant (Adamek et al., 2017).

*FOXP3* variants are associated with susceptibility to autoimmune diseases and infections (Fodor et al., 2011; Kwon et al., 2017), but the underlying mechanisms are poorly understood. The *FOXP3* c.-23+2882A>C variant can cause loss of binding of transcriptional factors to the “GGGCGG” sequence located in the core of the promoter region, altering the transcription of the gene (Oda et al., 2013). Functional analysis demonstrated the A allele of *FOXP3* c.-23+2882A>C decreased the promoter activity (Shen et al., 2010), however, this finding is not reliable since the article was retracted. Therefore, more studies are needed in order to clarify whether the *FOXP3* polymorphism behaves as a risk or protective factor for renal function.

Interestingly both *FOXP3* c.-22-902A>G and c.-23+2882A>C were associated with long-term gastrointestinal adverse effects, but this association was not maintained in the multivariable analysis after *p*-value adjustment, even when the patients were analyzed with the additive model (data not shown). To our knowledge, there is no report investigating the effect of *FOXP3* variants in the occurrence of adverse events.

Likewise, an association between the C allele of *FKBP1A* n.259+24936T>C and constipation was also found but this relationship was not sustained after Holm’s p-value adjustment. Moreover, *FKBP2* c.-2110G>T was associated with adverse event (leukopenia) but not with renal dysfunction or acute rejection in this sample population. However, this relationship did not remain after p-value adjustment in the multivariable logistic regression model.

Both MPS-based immunosuppressive therapy and ganciclovir were also individually associated with leukopenia, but only the latter remained significant after adjustment. It is likely that carriers of the GG genotype of *FKBP2* c.-2111G>T are more responsive to immunosuppressive drugs. Additional analysis showed that the GG genotype and the use of ganciclovir were not associated (data not shown). This might imply that CMV treatment is a strong predictor for leukopenia events rather than the *FKBP2* c.-2110G>T variant.

It has been described that up to 35% of patients under treatment with MPS develops episodes of leukopenia (Yang et al., 2015). Another factor causing this drug adverse effect is the use of ganciclovir (Matsumoto et al., 2015). Thus, individuals with susceptible genotypes could be exposed to a higher risk of leucopenia, which is favorable to the effect of immunosuppressive agents.

In our work, among the non-genetic factors influencing kidney outcomes, the time of DGF was strongly associated with acute rejection, even in the presence of other confounders such as recipient and donor factors or cold ischemia time. Likewise, in a cohort of kidney recipients treated with TAC-based therapy, the presence of DGF was associated with increased risk of acute rejection (Wu et al., 2015). These findings emphasize careful surveillance of patients with DGF in order to mitigate possible episodes of acute rejection.

To best of our knowledge, this is the first study to investigate the association of *MTOR*, *FKBP1A*, *FKBP2* and *FOXP3* polymorphisms with renal function, acute rejection and drug-related adverse events in Brazilian kidney recipients.

The small sample size and the population substructure are limitations of this study, and in particular, the low number of female patients for the analysis of polymorphisms located in the X chromosome. The low number of cases with acute rejection and graft failure also limited the study of associations between these outcomes and polymorphisms. Also a heterogeneity in the therapeutic regimens of calcineurin and mTOR inhibitors for every study group is observed. The analyses of this work are exploratory considering that the main study was not designed to evaluate pharmacogenetic aspects.

## Conclusion

In conclusion, this work shows that *MTOR* variants are associated with long-term kidney outcomes, in particular, with acute rejection and renal function. Furthermore, the variants in *FKBP2*, *FKBP1A*, and *FOXP3* were individually associated with the occurrence of drug-related adverse events.

## Author Contributions

AC-S, FG, AC, and RH wrote the article. AC-S, CF, HT-S, JM-P, MH, and RH designed the research. AC-S, GM, and RB performed the research. AC-S, FG, AC, and RH analyzed the data.

## Conflict of Interest Statement

The authors declare that the research was conducted in the absence of any commercial or financial relationships that could be construed as a potential conflict of interest.

## Abbreviations

Co/D: drug concentration for dose administered
CKD: chronic kidney disease
CMV: cytomegalovirus
CNI: calcineurin inhibitor
DGF: delayed graft function
eGFR: estimated glomerular filtration rate
EVR: everolimus
MPS: mycophenolate sodium
mTOR: mechanistic target of rapamycin
mTORi: mTOR inhibitor
TAC: tacrolimus
Tregs: regulatory T cells.
