# Supplementary material for: Polymorphisms in mTOR and Calcineurin Signaling Pathways Are Associated With Long-Term Clinical Outcomes in Kidney Transplant Recipients
Source: Front Pharmacol. 2018 Nov 14;9:1296. doi: 10.3389/fphar.2018.01296 (PMC6246626; doi:10.3389/fphar.2018.01296)
Supplement: Supplementary file 1 [file Data_Sheet_1.pdf]

Campus-Salazar et al. Frontiers in Pharmacology. 2018

Supplementary material

Suppl. Table 1. Drug monitoring data of kidney recipients at one-year post-transplant

Variables	TAC5/EVR (80)	TAC10/EVR (96)	TAC10/MPS (93)	*p-Value*
**Tacrolimus**				
Concentration, ng/mL	4.5 [3.7 – 5.8][a]	4.7 [3.4 – 6.4][a]	5.8 [4.4 – 7.2][b]	<0.0001
Dose, mg/kg	3.0 [2.0 – 4.0][a]	3.0 [2.0 – 6.0][ab]	4.0 [3.0 – 6.0][b]	0.007
Co/D, ng/mL*mg	1.53 [0.93 – 2.35]	1.52 [1.00 – 2.34]	1.37 [0.98 – 2.13]	0.939
**Everolimus**				
Concentration, ng/mL	6.2 [5.1 – 7.5]	5.6 [4.6 – 6.8]	-	0.073
Dose, mg/kg	4.0 [3.0 – 4.0]	4.0 [3.0 – 4.1]	-	0.857
Co/D, ng/mL*mg	1.80 [1.40 – 2.65]	1.53 [1.15 – 2.40]	-	0.177

Continuous variables are shown as median and interquartile range. Tacrolimus monitoring data were compared by Kruskal-Wallis test followed by Bonferroni's correction for multiple comparisons. Different superscript letters indicate significant differences. Everolimus monitoring data were compared by Mann-Whitney test. Co/Do, adjusted concentration for dose administered.

Suppl. Table 2. Association of gene polymorphisms with high serum creatinine in kidney recipients at one-year post-transplant: Univariable logistic regression analysis

**Polymorphism**	**Genotype**	**OR (95%CI)**	***p-Value***
MTOR c.1437T>C	CC<br>CT+TT	1.74 (0.96 – 3.15)<br>Reference	0.067
MTOR c.2997C>T	TT<br>TC+CC	1.30 (0.73 – 2.30)<br>Reference	0.371
MTOR c.4731G>A	AA<br>AG+GG	0.90 (0.50 – 1.62)<br>Reference	0.726
PPP3CA c.249G>A	GG<br>GA+AA	0.86 (0.46 – 1.62)<br>Reference	0.646
FKBP1A n.259+24936T>C	TT<br>TC+CC	0.94 (0.60 – 1.49)<br>Reference	0.807
FKBP2 c.-2110G>T	GG<br>GT+TT	1.19 (0.73 – 1.94)<br>Reference	0.475
FOXP3 c.-22-902A>G	GG<br>GA+AA	1.41 (0.89 – 2.23)<br>Reference	0.141
FOXP3 c.-23+2882A>C	CC<br>CA+AA	1.75 (1.07 – 2.86)<br>Reference	0.025

Analysis of MTOR polymorphisms was carried out with data from TAC5/EVR and TAC10/EVR groups. Serum creatinine was categorized in three groups (tertiles). The highest tertile (>1.50 mg/dL) was considered the risk group. OR: odds ratio; CI: Confidence interval.

Suppl. Table 3. Clinical variables associated with high serum creatinine in kidney recipients at one-year post-transplant: Univariable logistic regression analysis

**Independent variables**	**Risk factor**	**OR (95%CI)**	***p-Value***
**Model 1**			
Age	Per 1 year	0.99 (0.97 – 1.01)	0.530
Weight	Per 1 kg	1.03 (1.01 – 1.05)	0.012
Gender	Male	3.47 (1.87 – 6.46)	0.0001
Donor type	Deceased	0.85 (0.47 – 1.56)	0.602
Time on hemodialysis	Per 1 month	1.00 (0.99 – 1.01)	0.509
Delayed graft function time	Per 1 day	1.02 (0.98 – 1.06)	0.407
Cold ischemia time	Per 1 h	0.99 (0.97 – 1.02)	0.692
Acute rejection	Presence	3.21 (1.28 – 8.05)	0.013
Proteinuria	Presence	1.98 (0.79 – 4.95)	0.145
CMV	Use of ganciclovir	1.06 (0.35 – 3.22)	0.921
**Model 2**			
Age	Per 1 year	0.99 (0.97 – 1.00)	0.121
Weight	Per 1 kg	1.03 (1.01 – 1.05)	0.002
Gender	Male	4.00 (2.38 – 6.74)	0.0001
Donor type	Deceased	0.81 (0.50 – 1.31)	0.381
Time on hemodialysis	Per 1 month	1.00 (0.99 – 1.01)	0.843
Delayed graft function time	Per 1 day	1.02 (0.99 – 1.06)	0.206
Cold ischemia time	Per 1 h	0.99 (0.97 – 1.02)	0.612
Acute rejection	Presence	3.41 (1.63 – 7.10)	0.001
Proteinuria	Presence	1.88 (0.86 – 4.12)	0.112
Therapy group*	Presence	1.61 (0.99 – 2.62)	0.055
CMV	Use of ganciclovir	1.08 (0.58 – 1.99)	0.808

*Model 1: TAC5/EVR and TAC10/EVR groups. Model 2: All therapy groups. Serum creatinine was categorized in three groups (tertiles). The highest tertile (>1.50 mg/dL) was considered the risk group. OR: odds ratio; CI: Confidence interval. *Reference: TAC/EVR therapy group.*

Suppl. Table 4. Variables associated with acute rejection in kidney recipients at one-year post-transplant: Univariable regression analysis

**Independent variables**	**Risk Factor**	**OR (95%CI)**	***p-Value***
**Polymorphisms**	Genotype		
MTOR c.1437T>C	CC	Reference	
	CT+TT	1.25 (0.51 – 3.09)	0.609
MTOR c.2997C>T	TT	Reference	
	TC+CC	1.07 (0.45 – 2.55)	0.859
MTOR c.4731G>A	AA	Reference	
	AG+GG	3.37 (1.10 – 10.30)	0.037
PPP3CA c.249G>A	GG	Reference	
	GA+AA	0.82 (0.30 – 2.24)	0.681
FKBP1A n.259+24936T>C	TT	Reference	
	TC+CC	0.95 (0.48 – 1.86)	0.904
FKBP2 c.-2110G>T	GG	Reference	
	GT+TT	1.39 (0.66 – 2.93)	0.382
FOPX3 c.-22-902A>G	GG	Reference	
	GA+AA	1.14 (0.58 – 2.23)	0.735
FOXP3 c.-23+2882A>C	CC	Reference	
	CA+AA	0.88 (0.43 – 1.80)	0.725
**Other variables**			
Model 1			
Age	Per 1 year	0.98 (0.95 – 1.01)	0.139
Weight	Per 1 kilo	1.03 (0.99 – 1.06)	0.074
Donor type	Deceased	0.74 (0.33 – 1.67)	0.466
Time on hemodialysis	Per 1 month	1.00 (0.99 – 1.02)	0.485
Delayed graft function time	Per 1 day	1.06 (1.03 – 1.10)	0.001
Cold ischemia time	Per 1 hour	1.00 (0.96 – 1.04)	0.880
Model 2			
Age	Per 1 year	0.98 (0.95 – 1.00)	0.077
Weight	Per 1 kilo	1.01 (0.98 – 1.03)	0.485
Donor type	Deceased	0.91 (0.45 – 1.88)	0.807
Time on hemodialysis	Per 1 month	1.00 (0.99 – 1.01)	0.932
Delayed graft function time	Per 1 day	1.11 (1.06 – 1.16)	0.0001
Cold ischemia time	Per 1 hour	1.01 (0.97 – 1.04)	0.712

Analysis for MTOR polymorphisms was carried out with data from TAC5/EVR and TAC10/EVR groups. Other gene polymorphisms and other variables were analyzed using data from all therapy groups. Model 1: TAC5/EVR and TAC10/EVR groups. Model 2: All therapy groups. OR: odds ratio; CI: Confidence interval.

Suppl. Table 5. Variables associated with secondary clinical outcomes in kidney recipients at one-year post-transplant: Univariable logistic regression

Independent variables	Risk factor	OR (95%CI)	*p-Value*
**Leukopenia**			
FKBP2 c.-2110G>T	GG genotype	3.87 (1.26 – 11.91)	0.018
Age	Per every 1 year	1.03 (0.99 – 1.07)	0.122
Weight	Per every 1 kg	0.97 (0.93 – 1.01)	0.113
Gender	Male	0.92 (0.30 – 2.82)	0.878
Therapy group*	TAC+MPS	3.66 (1.19 – 11.28)	0.024
CMV	Use of ganciclovir	9.68 (3.08 – 30.38)	0.0001
**Constipation**			
FKBP1A n.259+24936T>C	TC+CC genotype	2.40 (1.08 – 5.37)	0.033
Age	Per every 1 year	1.01 (0.99 – 1.04)	0.425
Weight	Per every 1 kg	1.00 (0.97 – 1.03)	0.986
Gender	Male	2.15 (0.90 – 5.14)	0.087
Therapy group*	TAC+MPS	1.59 (0.77 – 3.30)	0.213
**Epigastric pain**			
FOXP3 c.-22-902A>G	GA+AA genotype	2.15 (1.01 – 4.56)	0.047
Age	Per every 1 year	1.01 (0.98 – 1.04)	0.428
Weight	Per every 1 kg	1.01 (0.98 – 1.04)	0.468
Gender	Male	0.83 (0.38 – 1.83)	0.648
Therapy group*	TAC+MPS	1.69 (0.81 – 3.53)	0.164
**Nausea and/or vomiting**			
FOXP3 c.-23+2882A>C	CA+AA genotype	2.38 (1.05 – 5.38)	0.038
Age	Per every 1 year	1.03 (1.00 – 1.06)	0.038
Weight	Per every 1 kg	1.01 (0.98 – 1.04)	0.587
Gender	Male	2.12 (0.94 – 4.78)	0.071
Therapy group*	TAC+MPS	1.00 (0.43 – 2.35)	0.996

**Reference: TAC+EVR therapy group. OR: odds ratio; CI: confidence interval; CMV: cytomegalovirus.*

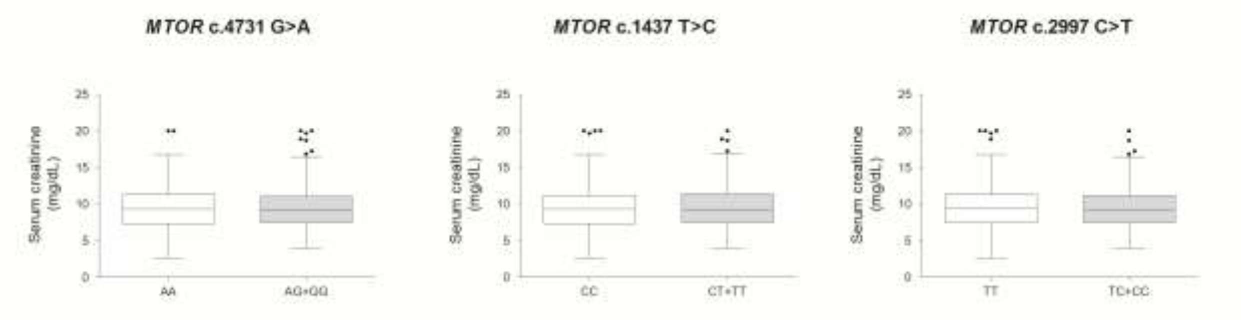

SUPPL. FIGURE 1. Influence of *MTOR* polymorphisms on pre-transplant serum creatinine levels in kidney recipients. Data are shown as box plots and interquartile range and compared by Mann-Whitney test. P>0.05.

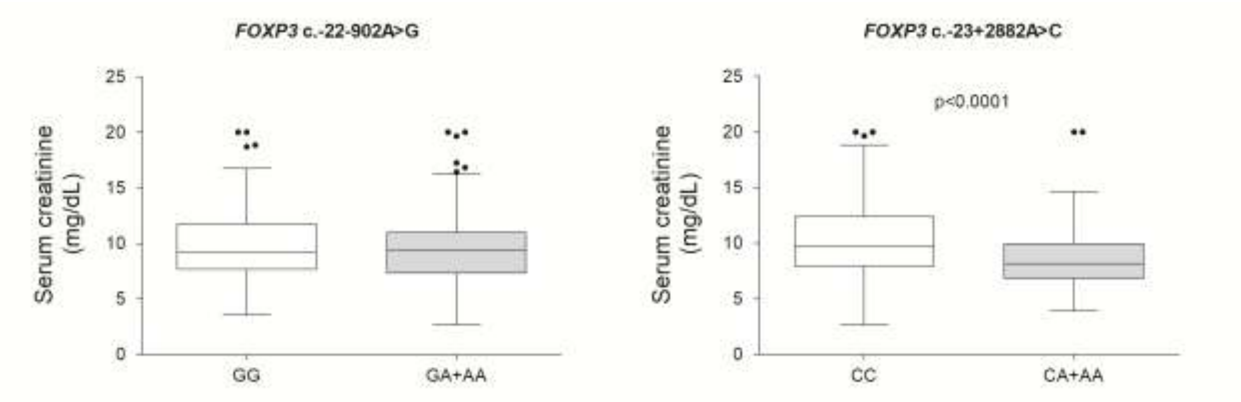

SUPPL. FIGURE 2. Influence of *FOXP3* polymorphisms on pre-transplant serum creatinine levels in kidney recipients. Data are shown as box plots and interquartile range and compared by Mann-Whitney test.
